# Supplementary figures and images for: Identification of Cytoplasmic Chaperone Networks Relevant for Respiratory Syncytial Virus Replication
Source: Front Microbiol. 2022 May 9;13:880394. doi: 10.3389/fmicb.2022.880394 (PMC9125393; doi:10.3389/fmicb.2022.880394)

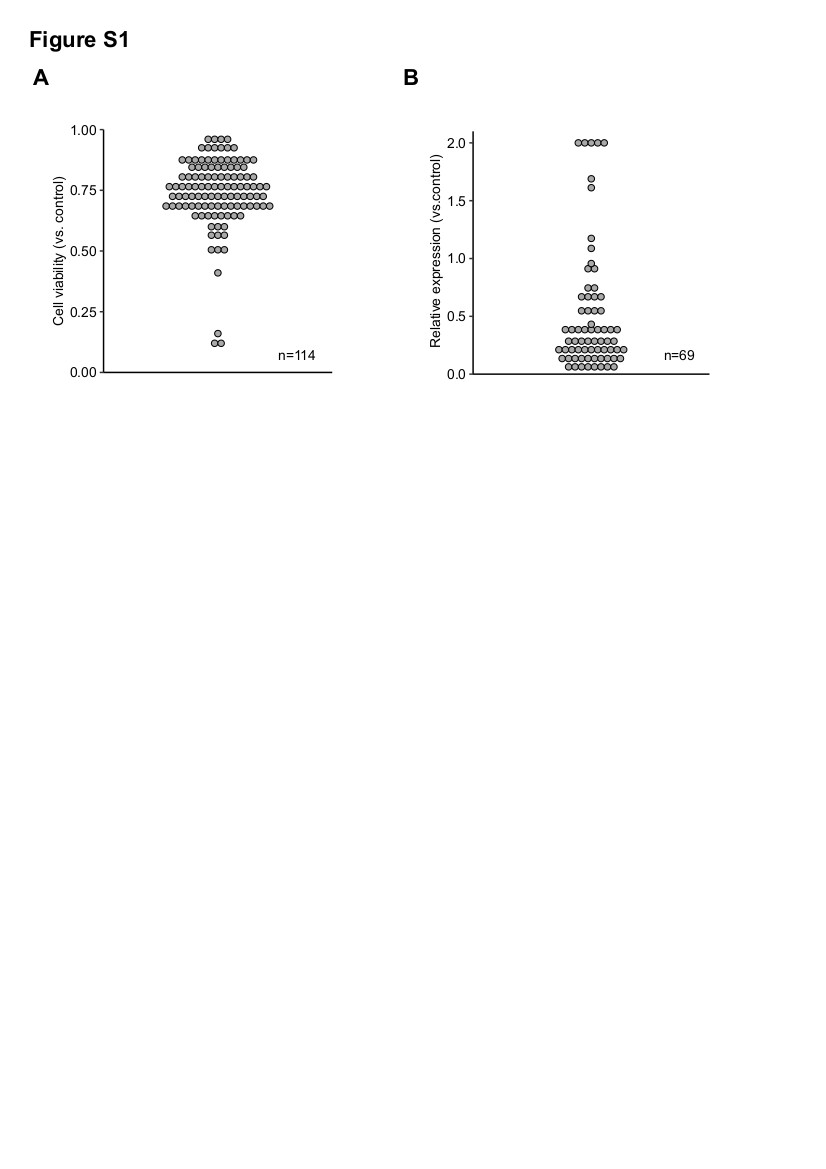

Supplement: Supplementary Figure 1 — Evaluation of the toxicity and knockdown efficiency for the esiRNAs used in the primary screen. (A) The relative viability of cells transfected with esiRNAs targeting cellular proteostasis components was compared to that of cells transfected with non-targeting esiRNA. (B) The expression of the targeted genes following esiRNA transfection was compared to that in cells transfected with non-targeting esiRNA. [file Image_1.JPEG]
